# Performance Measurement System and Quality Management in Data-Driven Industry 4.0: A Review

**DOI:** 10.3390/s22010224

**Published:** 2021-12-29

**Authors:** Parkash Tambare, Chandrashekhar Meshram, Cheng-Chi Lee, Rakesh Jagdish Ramteke, Agbotiname Lucky Imoize

**Affiliations:** 1 Water Resources & Applied Mathematics Research Lab, Nagpur 440027, Maharashtra, India; prakash.tambare058@gmail.com; 2 Department of Post Graduate Studies and Research in Mathematics, Jaywanti Haksar Govt. Post-Graduation College, College of Chhindwara University, Betul 460001, Madhya Pradesh, India; 3 Department of Library and Information Science, Research and Development Center for Physical Education, Health, and Information Technology, Fu Jen Catholic University, New Taipei 24205, Taiwan; 4 Department of Computer Science and Information Engineering, Asia University, Wufeng Shiang, Taichung 41354, Taiwan; 5 School of Computer Sciences, KBC North Maharashtra University, P.B. No.80, Umavinagar, Jalgaon 425001, Maharashtra, India; rakeshramteke@yahoo.co.in; 6 Department of Electrical and Electronics Engineering, Faculty of Engineering, University of Lagos, Akoka, Lagos 100213, Nigeria; aimoize@unilag.edu.ng; 7 Department of Electrical Engineering and Information Technology, Institute of Digital Communication, Ruhr University, 44801 Bochum, Germany

**Keywords:** Industry 4.0, Internet of Things, Quality 4.0, performance measurement system, cyber–physical production system

## Abstract

The birth of mass production started in the early 1900s. The manufacturing industries were transformed from mechanization to digitalization with the help of Information and Communication Technology (ICT). Now, the advancement of ICT and the Internet of Things has enabled smart manufacturing or Industry 4.0. Industry 4.0 refers to the various technologies that are transforming the way we work in manufacturing industries such as Internet of Things, cloud, big data, AI, robotics, blockchain, autonomous vehicles, enterprise software, etc. Additionally, the Industry 4.0 concept refers to new production patterns involving new technologies, manufacturing factors, and workforce organization. It changes the production process and creates a highly efficient production system that reduces production costs and improves product quality. The concept of Industry 4.0 is relatively new; there is high uncertainty, lack of knowledge and limited publication about the performance measurement and quality management with respect to Industry 4.0. Conversely, manufacturing companies are still struggling to understand the variety of Industry 4.0 technologies. Industrial standards are used to measure performance and manage the quality of the product and services. In order to fill this gap, our study focuses on how the manufacturing industries use different industrial standards to measure performance and manage the quality of the product and services. This paper reviews the current methods, industrial standards, key performance indicators (KPIs) used for performance measurement systems in data-driven Industry 4.0, and the case studies to understand how smart manufacturing companies are taking advantage of Industry 4.0. Furthermore, this article discusses the digitalization of quality called Quality 4.0, research challenges and opportunities in data-driven Industry 4.0 are discussed.

## 1. Introduction

Recent technological innovation is evolving rapidly due to emerging technologies such as artificial intelligence (AI), Internet of Things (IoT), cloud computing, machine learning (ML), big data, and the manufacturing industries [1,2,3,4]. These stage technologies permeate the production process to make the industry smart enough to address current challenges such as increased personalized requirements, increased quality, and reduced production cost. Others include offering effective solutions, serving customers with efficiency, speed, cost/benefit, higher performance, and reduced time to market [3,5,6]. Digitalization, automation and adaptation, optimization and production customization, human–machine interaction (HMI), value-added services and businesses, digital data exchange, and collaboration are the five major components of Industry 4.0 [7,8,9]. The factory operator’s functional paradigm has changed from physical exertion to cognitive workload, with successive industrial revolutions in the last few decades due to the rise in use of ICT in factory automation and the sophistication of information [6,10,11].

In Industry 1.0, the first revolution began in 1784 and was marked by steam power and mechanization. The single operator was deployed in Industry 1.0 to supervise and control the entire manufacturing process from the electromechanical dials connected locally to machines in the factory. The operator had to move around the factory to track the production processes and machine statuses to gather all the knowledge about the process and equipment working in the manufacturing facility. The second industrial revolution, also called Industry 2.0, occurred at the end of the 19th century with the invention of electrification factories. The electrification of factories permitted continuous round-the-clock operation, mass production, and process parameters controlled from isolated control rooms. In Industry 3.0, the computer was introduced for the manufacturing industries to automate the manufacturing processes. Computer-based production processes and systems are implemented into manufacturing activities and include several devices (i.e., programmable logic controller and supervisory and data acquisition systems). Robots are used in some operations to control the production process remotely [1,6,10].

The fourth industrial revolution is called Industry 4.0, the German government strategy group’s name. Industry 4.0 is attracting the attention of researchers and practitioners globally [12,13]. The key focus of Industry 4.0 is on emerging technology that will have a huge effect on production processes. These innovations include virtual reality, 3D printing, simulation, big data analytics, cloud computing, radio frequency identification, Internet of Things, cybersecurity, machine-to-machine communication, robots, drones, nanotechnology, and business intelligence (BI) [14,15,16]. These will radically alter manufacturing processes and can be tailored to customer requirements. Moreover, these new technologies, particularly the IoT and cyber–physical systems (CPS), will impact products and services, markets, business models, the economy, work environment, human and business capabilities, and profoundly transform production processes [1,6,12,15,17].

Industry 4.0 uses the Internet of Things (IoT) to develop a cyber–physical production system (CPPS). The CPPS is envisioned as the core technology of Industry 4.0, which will comprise technologies such as IoT, wireless embedded network systems and network cloud computing, big data, and AI in manufacturing plants [6]. The study found that Industry 4.0 enables digital factories to deliver more competitive advantages than traditional manufacturing [18]. The CPPS allows the exchanging of production data over the internet with multiple systems in the smart factory. The use of software and advanced computer technology has led to merging physical (machines, sensors, actuators, etc.) and virtual (cloud, AI, ML, big data, IoT, wireless Communication, etc.) worlds, which is called a cyber–physical System [11,19,20,21] Industry 4.0, and it will bring information technology and factory automation together to produce smart manufacturing [22,23].

Smart manufacturing is fully integrated, and collaborative manufacturing frameworks can respond to evolving requirements and conditions in the factory, supply networks, and consumer needs in real-time [24,25].

Industry 4.0 opportunities can be broken down into the following key fields: performance flexibility that happens during small-batch manufacturing; the speed of serial prototypes; production capacity; minimized setup cost, fewer errors and low machines downtime; increased product quality and less rejected production; and improved consumer opinion on products [2].

The importance of more detailed mechanisms for performance assessment schemes was widely discussed in the 1990s. The performance measurement concept began to be consolidated, and significant contributions were made, including performance images [17]. Managing a production facility, including product quality, machine efficiency, and overall performance, has become essential to the manufacturing industry for the effective processing of products and product quality. The production plant manager will assess the key performance indicators (KPIs) used to measure the machine’s performance, the overall production process, or the part of the production process [26]. The performance measurement matrices are critical parameters in the production plant because well-defined KPIs allow us to find the performance gaps between the current and desired operations, which can monitor the progress toward closing the gaps [27] in today’s data-driven manufacturing industries. Performance monitoring and quality control are critical for growing the efficiency and quality of their processes and products to face the competitive market. In this article, we have drawn the concepts from multiple disciplines to present the methodology for implementing the key performance indicators (KPIs) defined in ISO 22400 standard-automation systems and integration [27,28,29], ANSI/ISA-95 standards for Integrating MES and ERP Systems [27,30,31,32,33,34].

Quality is a fundamental feature of products and processes in any manufacturing industry. For businesses and organizations in the global market, this is considered a strategic advantage. In modern history, quality models and practices have undergone many evolutionary phases, from inspection to control, monitoring, quality assurance, quality management, and design quality. These quality models are a function of industry trends and developments. After a few years of stagnation in rate, few creative quality models are being proposed, and quality professionals’ leadership roles in businesses and organizations seem to have faded. Furthermore, there is no research into modern and creative quality models. The fourth industrial revolution is an opportunity for the quality movement to become a leading power [33,35].

**Figure 1:**
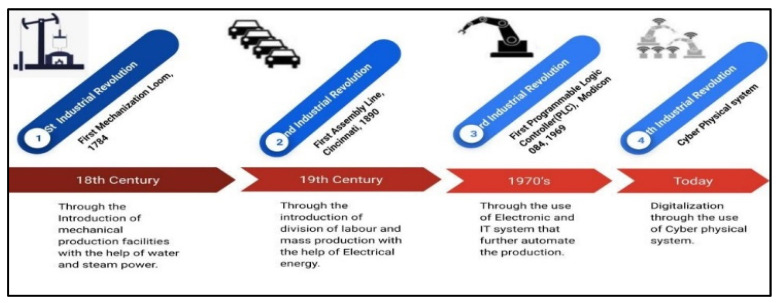
Industry 1.0 to 4.0.

Figure 1 illustrates the successive technological revolutions as how people and machines communicate. They have changed from the first Industrial revolution to the fourth Industrial revolution. The concepts that make up the term Quality 4.0 were predicted more than 20 years ago due to the increasing availability of telecommunications technology, the internet, personal computers, networks, and machine learning schemes that can somehow perform quality functions and analysis automatically [36,37]. Quality 4.0 refers to Industry 4.0 to enhance quality through smart solutions and smart algorithms [38,39,40]. This topic is too fresh, and therefore, discussions and knowledge sharing are primarily conducted through research papers [41]. Quality 4.0 studies are currently being undertaken by many firms such as LNS Consulting Group. According to its inquiries, most manufacturing firms will have to convert to Industry 4.0 within the next five years, including quality control transfer [39,42]. The LNS Research group-based Quality Management and Quality 4.0 defined using case studies to understand how the smart manufacturing industries adopt the standards and apply these standards in their initiatives to benefit from Industry 4.0 [19,43,44,45,46]. There is growing interest in Industry 4.0, but there is a lack of detailed reviews on performance measurement and quality management in data-driven Industry 4.0. This paper explores the tools, methods, and industry standards used in smart factories to measure performance and manage quality. Furthermore, it discusses Industry 4.0’s research challenges and opportunities.

The analysis is carried out with three research questions in mind: (1) What are the various methods, tools, and standards used to measure the performance of Industry 4.0? (2) What are the different approaches and techniques used to manage the quality of the products in Industry 4.0? (3) What are the current challenges and opportunities in Industry 4.0? [47,48,49,50].

### 1.1. Problem Statement

The evolution of manufacturing is already on its path to “Industry 4.0”. According to the findings, the Industry 4.0 initiative will have high demand in the future. As the concept of Industry 4.0 is relatively new, there is high uncertainty, lack of knowledge and limited publication about the performance measurement and quality management with respect to Industry 4.0.

Conversely, manufacturing companies are still struggling to understand the variety of Industry 4.0 technologies. Industrial standards are used to measure the performance and manage the quality of the product and services. In order to fill this gap, our study focuses on how the manufacturing industries are using different industrial standards to measure the performance and manage the quality of the product and services [48,51,52,53,54].

### 1.2. Motivation

The rapid change in ICT development impacts most of the manufacturing industries. Several CEOs of the manufacturing industries worldwide are thinking about implementing the Industry 4.0 concept and have many real-time questions that need to be addressed. How do we measure the performance? What are the KPIs that need to be set, and what standards need to be adopted to measure the performance of Data-Driven Industry 4.0? The most crucial part of the manufacturing industry is Performance and Quality measurement. The rapid advancement of ICTs has changed the paradigm of industries operation [47,48]. The two factors that motivate the research undertaken in this review paper are as follows. First, we look at the performance measurement in Data-Driven Industry 4.0 and the Quality measurement System in Industry 4.0.

### 1.3. Contribution

Manufacturing Sectors are in a constant transition state, with the digitalization and innovation of ICTs. It is becoming a big challenge for industries to stay on the market. Big data, automation, AI, IoT, and cloud computing in the research community are widely discussed. Although there is research on performance assessment and quality management systems, it has been developed primarily in a stable environment. This study demonstrates how the latest research focuses on implementing the performance and quality measurement criteria in Data-Driven Industry 4.0, where different industrial standards are used to assess the performance and quality of Industry 4.0.

### 1.4. Organization

The paper is organized as follows. The review methodology used for the SLR is illustrated in Section 2. The methods are discussed in Section 3, which includes performance measurement and quality management and the case studies. Section 4 provides the scope of research challenges, opportunities, and the scope of future work. Section 5 concludes the analysis and presents the research contributions and shortcomings of the research.

## 2. Literature Review

Ramamurthy and Jain [10] addressed the idea of Industry 4.0, the Internet of Things, cyber–physical Production System. Recent developments in ICT, such as artificial intelligence, machine learning, big data, the Internet of Things, and cloud computing, allow intelligent and highly reconfigurable factories to be developed, leading to unprecedented output growth. One of the cornerstones of what is considered to be the fourth Industrial Revolution is the notion of the Cyber–Physical Production System (CPPS). In this system, the mechatronic components are smart, allowing the factory units to communicate adaptively [19,47,48,55].

There were substantial productivity gains in the previous three revolutions: first, steam and water, electricity and assembly lines, and then computerization. The Internet of Things uses the network and networking infrastructure to link the fourth industrial revolution to computers, devices, machines, and people [10,56]. The Institute of Electrical and Electronics Engineers (IEEE) defines IoT as follows: “An IoT is a network that connects uniquely identifiable ‘Things’ to the internet. ‘Things’ have sensing/actuation and potential programmability capabilities. Through the exploitation of unique identification and sensing, information about the ‘Thing’ can be collected. Additionally, the ‘Thing’ state can be changed from anywhere, anytime, by anything are broached [57]”.

In this article, the authors addressed the fourth industrial revolution; performance assessment of production systems in a network whose success is based on production system robustness. Efficient and reliable performance assessment can significantly impact an industrial company’s profitability [58]. The authors explored developing a method for systemic analysis of an IoT-based production model in line with ISA-95 and ISO 22400. These two principles explain how a production process can be formalized and how the performance metrics can be formalized. The authors have built a unified method to generate a smart factory performance measurement framework by applying the IoT data anomaly response model. In the case of IoT data failure, the IoT data anomaly response model is executed. Using a K-means clustering approach and a statistical method, the solution model’s goal is to identify an IoT data anomaly and minimize the effect of the IoT data anomaly. This research examines the link between expected and real abnormal output data based on the “Overall Equipment Effectiveness” [27]. The fourth industrial revolution’s adaptation causes a significant change in manufacturing processes today [19]. To help incorporate a cyber–physical system approach, the author describes metrics and methods and explains how to build a new Key Performance Indicator (KPI) in smart manufacturing based on ISO 22400. It also discusses the Scania case study to understand smart manufacturing performance indicators. The defined KPIs are the Operational Equipment Effectiveness and Process Capability Index (Cp, Cpk).

The performance assessment of production processes is ultimately driven by performance indicators or Key Performance Indicators (KPIs). KPIs are modern instruments that make it easier to maintain high performance in manufacturing [43]. In addition, performance metrics express what has happened; they show what will happen, as they provide the decision-maker with the knowledge that will influence the company’s future competitive position [59]. The roles of production performance indicators are to represent the current state of production, track and monitor operational quality, drive a change program, and measure strategic decision-making effectiveness [60]. Quality, cost, delivery time, and flexibility are the most widely cited metrics for measuring performance in production systems [1]. Modern information technologies allow quality management to be incorporated into technical processes and quality management in real-time [61].

## 3. Methodologies

Different industrial standard and case study approaches are used as a research tool to achieve and represent the proposed concept of performance and quality assessment of smart production systems. A literature review of the associated context was also conducted to explain and understand the main techniques to establish a performance and quality measurement concept in data Driven Industry 4.0. There are many KPIs used to measure the industry’s performance. Here in the methodologies section, we discuss different KPIs used in manufacturing plants at the shop floor production level and a case study to understand the performance evaluation in Industry 4.0. Further, we will discuss the Quality measurement approach and a case study concerning Industry 4.0. The Table 1 summarizes the performance measurement and quality management approaches used in the Section 3.

**Table 1:** Performance measurement and quality management approaches.

Ind. Std.	Performance Measurement Methodologies	Ind. Std	Quality Management Methodologies	Ref
	Different Industrial Standards and case studies used as a research tool.	LNS Framework	The LNS research defined 11 Axes of Quality 4.0 as a research framework along with the case studies as a research tool.	[30,62]
	ISA-95 and ISO 22400 standards are used to measure the performance.	LNS Framework	The LNS research defined 11 Axes of the Quality 4.0 Framework, which allows the company to implement Quality Management System.	[19,30,62,63,64]
ISA-95	Integration of Enterprise and control System Enterprise System is Information Technologies such as ERP, CRM, etc. Control System—Operation Technologies such as SCADA, PLC, Sensors, etc. The ANSI/ISA-95 standard is an automated interface between factory control systems and enterprise systems. The ISA-95 standard describes entities at the shop floor level, where IT (ERP, CRM, Could, SQL, etc.) and OT (Sensors, Actuates, Microcontrollers, SCADA, PLCs, etc.) interact.	LNS Framework	Data: The data are the key element in the new quality paradigm. Analytics: Industry 4.0’s advanced technologies enable us to gather massive data from the production plant and apply the analytics tools to measure quality matrices. Connectivity: Enables data to flow between systems, which allows organizations to improve the quality of their products and services. Collaboration: Quality 4.0 leverages modern technology—such as social listening and blocking to analyze factors such as customer satisfaction, component supply, and distribution across supply chains.	[19,22,31,65]
ISO 22400	Creating a key performance indicator (KPI) means that the result and the performance of the targets can be shown. The KPI allows tracking progress and displaying it in a quantifiable form. ISO 22400 defines KPIs for smart manufacturing. KPIs are used to measure the performance ISO 22400 and ANSI ISA-95 work together to define the KPIs KPI-ML is an XML version of ISO 22400, which is being used in smart manufacturing. KPI-ML is used to record, interact and exchange the KPI Knowledge. The details ISO 22400 KPI description Table 2. These KPIs require data from several processes and machines. The details of most Common KPIs used in industry is shown in Table 3.	LNS Framework	App Development: Helps to improve services by collecting users’ feedback and essential information. Scalability: Industry 4.0 technologies are the tools that allow companies to grow at a quicker pace. Management System: Improving system autonomy reduces high-value workers and managers’ time on implementation, encouraging them to focus on improved and innovative jobs. Compliance: Data collection tasks relating to regulation can be automated by integrating IT and OT. Culture: Quality 4.0 connects data, analytics, and processes to improve visibility, connectivity with other departments and provide a corporate culture that values quality. Leadership: Quality 4.0 creates the right quality culture throughout the organization. Competency: Quality 4.0 encapsulates several innovations that can be used to enhance competency.	[19,30,62,63,64,66]
Scania Case study	The ISO 22400 standards is used to implement KPIs to measure the performance in Scania Pedal Car Line. The Scania Pedal Car Line uses sophisticated technology and intelligent resources recently updated from advanced tools—a smart device that can connect with other systems. The acquired data from the connected system is used to extract the KPIs to measure the performance. This can be formed in three steps Data Collection, Data Identification and Data Planning.	Rolls-Royce Case Study	Rolls-Royce is a producer of aircraft engines supplying more than 150 military aircraft engines and 500 airlines The manufacturing production plant of Rolls-Royce has been connected, and IoT technology has been applied; the organization uses advanced technologies such as big data to manage aircraft engines and generate a considerable amount of data. The Rolls-Royce Company collects data from various sources, such as design, manufacturing and post-sales management.	[19,63,64,67,68,69,70,71,72]
	Data Collection: The system is event-driven and sends the request to obtain the data and the requested information that the tool sent at the given time.		It analyzes this data and uses it to generate useful and predictive information for maintenance and quality operations.	[19,63,64,67,68,69,70,71,72]
	Data Identification: The standards allow for a common framework for metrics and measures. The resulting data are standardized, creating uniform definitions according to the ISO 22400 template. These well-defined metrics can then be obtained from the system.		Rolls-Royce offers a post-sale Total Care Service that provides real-time monitoring through data collection. Rolls-Royce can use comprehensive data analysis, intelligent sensors, AI, and platform construction to retain quality control by predictive maintenance.	[19,67,68,69,70]
	Data planning is collecting, preparing, analyzing and arranging data to be used for KPI analysis.		Rolls-Royce uses nanobots for predictive maintenance and inspections at the production plant.	[63,64,71,72]

Ind. Std.—Industrial Standards.

### 3.1. Performance Measurement System

The evolution of manufacturing is already on its path to “Industry 4.0.” According to the findings, the Industry 4.0 initiative will have high demand in the future [52,73,74] and it requires rethinking on how performance can be measured in Industry 4.0. Adaptation is essential because the Industry 4.0 setting differs from previous planning, operations, and management systems [67]. The performance of the production plant can be enhanced using the technologies of Industry 4.0 [11,75].

In this section, we discuss how to measure performance in Industry 4.0. We use two different standards and case studies to understand how performance measurement is implemented in Industry 4.0. The first is the ISA-95 standard, and the second is the ISO 22400 standard. The International Standardization creates and provides “requirements, specification, guidelines or characteristics that can be used consistently to ensure that materials, product, process, and services are fit for their Purpose” [65]. We address the development by the American National Standard ANSI/ISA-95 of an automated interface among control systems and enterprise systems found in factories [19,30,31,32].

The ISA-95 standard describes entities at the shop floor level, where Information technologies (ERP, CRM, Could, SQL, etc.) and Operation Technologies (Sensors, Actuates, Microcontrollers, SCADA, PLCs, etc.) interact [55,76,77,78,79] and the International Organization for Standardization ISO 22400 is a standard describing KPIs in manufacturing [19,28,29,80]. It focuses on performance measures that serve as the foundation for achieving continuous operational performance improvement in manufacturing through key performance indicators (KPIs) based on various measurements derived from the context of the operation [81,82,83]. Smart manufacturing standards are important to ISO, ANSI/ISA-95, and IEC [80].

#### 3.1.1. ISA-95

**Figure 2:**
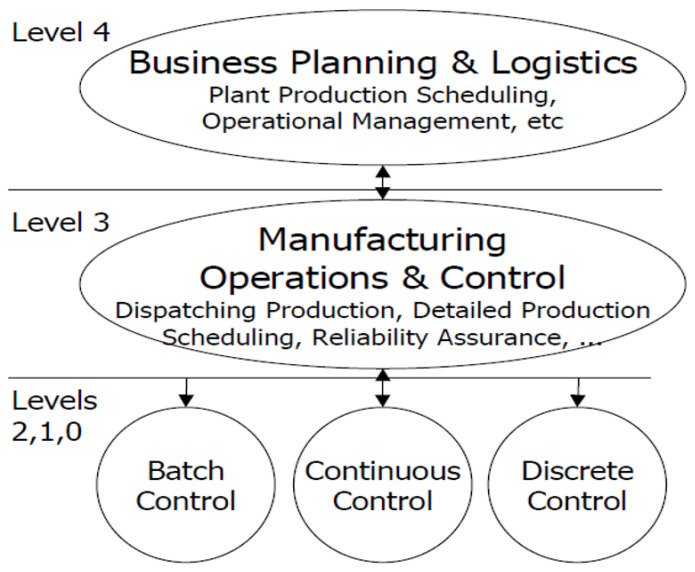
Functional hierarchy of production as specified in ISA-95.

The American National Standard ANSI/ISA-95 [30,62,79] contains standards defining various production and automation components. The bar is entitled “Integration of the Enterprise-Control System” [30,62], and the title reads as how to incorporate enterprise/business systems with production and control systems [65]. Figure 2 represents the functional hierarchy of production described in ISA-95 based on the Purdue Enterprise Reference Architecture. Level 0 represents the physical and industrial processes such as sensors, level 1 represents sensors and actuators’ roles, and level 2 represents monitoring and process control. Level 3 represents the manufacturing activity and control, such as the workflow that processes the final product, maintains the records, and coordinates the processes. Business planning and logistics refer to level 4, where plant production scheduling and operations management are performed. Information from level 3 is vital for level 4 functions [19,30,62,65,84].

- Manufacturing Operation Center (MOC) Using ISA 95

This section will understand the concept and working principle of MOC, which is discussed in [43,77]. The team of Oracle Inc. developed Manufacturing Operation Center (MOC) using ISA 95 Standards. The MOC provides a solution to the manufacturing plants to monitor and enhance plant performance by evaluating plant floor data in real-time. Manufacturing Operations Center provides manufacturers with real-time visibility into shop floor performance. MOC contextualizes shop floor data obtained from a variety of sensors, Programmable Logic Controllers (PLCs), Supervisory Control and Data Acquisition (SCADA), Distributed Control System (DCS), etc. These comprise enterprise system data and provide pre-built dashboards based on the ISA-95 reference model [43,77,81].

The MOC system meets the manufacturing plant’s needs by providing exact and timely information regarding the product, production quality, manufacturing processes, and asset performance. The MOC system solves the issue of production plants from the disconnected production floor data to the connected back-office system’s enterprise situation. This integration offers real-time monitoring and analysis of production floor activities [43,81].

The MOC system uses Fusion Middleware’s integration framework to collect data sources, including an MES application or a quality application. The key partners such as Kepware, ILS Technologies and Matrikon provide gateways to capture real-time data from plant equipment and control systems [85]. The Oracle Data Warehouse 10 g is processed and contextualized to offer plant managers and production supervisors the collected data as specific KPI on role-based dashboards [43].

The MOC framework utilizes a functional contextualization engine to identify business definitions and production process guidelines for numerous tag data obtained from PLCs and different automation devices. The collected data will be processed, and the processed data will be displayed on a dashboard that interprets the data at different organization levels. The MOC has 55 predefined KPIs, [19,43] and these KPIs displayed on 14 dashboards: (a) asset performance overall equipment effectiveness (OEE), (b) asset performance (OEE) by equipment, (c) equipment downtime analysis, (d) equipment downtime reasons, (e) production slippage pattern, (f) production loss analysis, (g) production loss information, (h) equipment efficiency analysis, (i) equipment scrap analysis, (j) equipment scrap reasons, (k) batch performance, (l) batch performance detail, and (m) production performance.

ii. Use case I: Overall Equipment Effectiveness (OEE) and production loss review

The initial use case of MOC is introduced to illustrate the Total Productive Maintenance (TPM). The initiative TPM describes a “synergistic relationship among all organizational functions, but particularly between production and maintenance, for the continuous improvement of product quality, operational efficiency, capacity assurance, and safety [86]. TPM attacks “six big losses,” draining efficiency consisting of breakdowns, loss of setup, idling/low stoppages, reduced speed, defect/rework, startup/render losses attacks [44,86].

To determine TPM initiatives, the elimination of these losses improves OEE’s most common numerical metric [31,32]

(1) OEE = availability × performance × quality

where

Availability = Actual available time/Planned available time;
Performance = Effective run time/Actual available time;
Quality = Good quantity produced/Total quantity produced.

Due to breakdowns, setups, and modifications, availability captures deleterious effects. The performance captures productivity loss due to lower pace, idling, lesser stoppages, and the suitable product yield that captures loss due to defects rework, and the result is quality.

The OEE calculations for a multi-site production company are shown on the different KPI dashboards. The plant manager will drill down to the equipment level to investigate the cause of low OEEE when the overall OEE is near the red area (above 75 per cent). The most inferior five performing devices will be analyzed and investigated to discover the root cause. If availability is a factory’s lowest OEE part, they can browse the factory equipment over each downtime cycle and examine reasons for downtime.

As seen in this use case, the advantages of ERP-level data integration with shop floor level are increased access to process performance measures and quality improvement by enabling an in-depth examination of the root cause of problems.

#### 3.1.2. ISO 22400

The International Organization for Standardization ISO 22400 [28,29,87] is a standard that specifies KPIs for manufacturing. The Table 2 shows an ISO 22400 KPI description.

The development and uniformity of a structured way of producing KPIs benefit the industry [19]. ISO 22400 and ANSI ISA-95 work together to define the KPIs in three sorts of MOM industries; batch, Continuous, and Discrete [88,89]. ISO 22400 sets the requirements for a KPI, and MESA International has produced KPI-ML, an XML version of the ISO 22400 Specification currently being used to record, interact, and exchange KPI knowledge [90]. ISO 22400 is a multinational, non-profit organization of production firms, IT manufacturers, systems integration, vendor consultancy, researchers, authors, academics, and students. To provide information that is crucial to understanding the KPI, KPI-ML extends the sharing of ISO 22400 data, including the values used for calculating the KPI [64,76,91].

**Figure 3:**
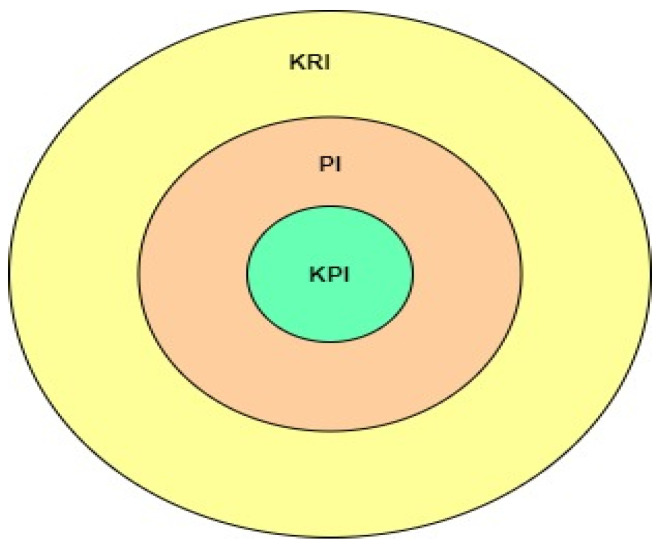
KPI onion mode.

This subsection describes the ISO 22400 standards and how to apply these standards in the industry to define the different KPIs to measure the various Smart manufacturing parameters’ performance and the most common KPIs used in the industry case study. An onion metaphor, see Figure 3, will explain the definition of KPI. If the onion center is the KPIs, the outer shell is the direct measurement called key result indicators (KRI). The KRI is collected from the machines, sensors, and equipment from the production plants to provide measurable results [19,63].

The second inner layers are known as performance indicators (PIs). These involve either a single KRI or a group in an equation. Both the KRIs and the PIs operate in cooperation with the KPIs. Creating a KPI means that the result and the performance of the targets can be shown and It is built to see what can be done to increase productivity and display it quantitatively. The Manufacturing Enterprise Solutions Association (MESA) investigation was conducted to see the industry’s most used KPIs [19,63]. The most common KPIs used in the industry are shown in Table 3.

These KPIs require data from several processes and machines. Acquiring this data in a cyber–physical system is many times simpler than traditional manufacturing sites due to the interconnected nature of cyber–physical systems. Another view of the most common KPIs is the visual process, which is critical to show in different departments [29]. These are shown in Table 4.

##### Test Case: Scania

In [19], the author explained and carried out the research work on the ISO 22400 standards to implement KPIs to measure the performance in Scania Pedal Car Line. The pedal car line in Scania is designed to use for different purposes and reflect those criteria. The pedal car system in Scania was used to experiment with the new machine, new tools, test the new system and their control system, and use it for showcase room for new innovative technologies.

Furthermore, it explains how to create a pedal car step by step on the assembly line, which involves both new pedal car assemblies and the disassembly of those already made; all this teaching requirement is the main workflow to be used in actual production. In this section, we reflect on the showcase part of new systems, in which staff members can see how Smart Factory processes the data. This section has the most sophisticated technology and intelligent resources recently updated from advanced tools—a smart device that can connect with other systems [15]. The first implementation involves connecting power tools to the ESB to obtain data from systems. The acquired data are used to extract the KPIs to measure the performance. cyber–physical systems with sensors and actuators which are now linked and communicate have an advantage over traditional methods. There was no automation in the pedal car line before. As such, all data flows have been historically manually carried out either by workers or paper [67].

Data acquirement/Acquisition: Atlas Copco designed the Power Focus PLC System, which provides different functionalities such as each controller’s status, communication, event monitoring, tightening, communication, Synchronization, API, Cell, etc., to automate the manufacturing process [92,93]. The power focus concept is a cell in which one graph can monitor and control 20 compact controllers. Each controller is connected to a network via the ethernet port and monitors the Atlas Copco TookNet Server [68]. The system is event driven and sends the request to obtain the data and the requested information sent by the tool at the given time. In the system, the received data are a long string with all the data bundled together. You can collect the desired information by dissecting this string. The tools used are for bolt tightening and have fast connectors for different bolts [94].

Data identification: The KPI calculation, as possible or not possible, cannot be decided by comparing the data at hand with what is required for the new KPI calculation. However, this is closely related to the understanding and implementation of the standards. The standards create a common framework for the metrics, and measures can only be derived from this basis, creating uniform definitions according to the ISO 22400 template for each data point. The results are well-defined metrics available from the system.

Data planning: Many KPI values will be calculated from the extracted data and analyzed with available matrices in the data planning. First pass yield, availability, throughput rate, downtime, OEE (time-based), scrap ratio, count, goal, and takt time are the KPIs. New KPI concepts have been made for the new KPIs following the specifications given by ISA-95 and ISO 22400.

### 3.2. Quality Management and Quality 4.0

Currently, the quality of products, services, and processes are crucial for achieving sustained economic development and maintaining productivity [9,37,93,95]. Quality control and management have attracted many scholars’ and managers’ interest, and it is an important area of study and research [23,46]. Manufacturers must transition to the “Quality 4.0” concept to integrate new technologies to analyze the data and assess quality [96,97]. Quality 4.0 is a term that refers to the increasing digitization of industry, which employs advanced technologies to improve the quality of manufacturing and services [25,40,98,99,100].

Quality 4.0 is a reference point for Industry 4.0 [50,99]. Quality 4.0 requires the digitalization of the management of quality. This digitalization of quality technology, processes, and people [101] is more significant. It builds on traditional quality equipment and considers collaboration, intelligence, and automation in an end-to-end scenario to boost efficiency, make timely data-driven decisions, involve all stakeholders, and provide visibility and accountability [40,102,103].

LNS defined 11 axes of quality 4.0 that organizations can use to teach, prepare, and act. Using this framework and study, leaders can define how Quality 4.0 will transform current skills and initiatives. The framework also offers a view of conventional consistency. Quality 4.0 does not replace traditional methods of quality but instead builds on and enhances them. Manufacturers should use the framework to interpret their current state and decide what improvements are required to transition to the future. Data-driven decisions have been at the center of quality management for decades. Many recently revised criteria stress the significance of evidence-based decision making [24,104].

#### The 11 Axes of Quality 4.0

**Figure 4:**
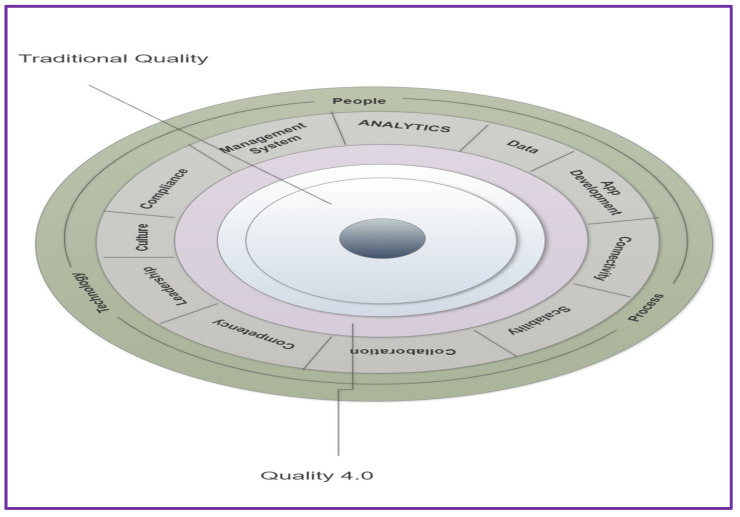
Eleven axes of Quality 4.0.

The LNS report defined 11 Axes of the Quality 4.0 Framework, which allows the company to implement Quality 4.0 due to the 4th industrial revolution Quality Management System. Here are the 11 Quality 4.0 Axes discussed below [70,92,101,104]. The LNS report defined 11 Axes of the Quality 4.0 Framework shown in Figure 4.

Data: Data have always played a critical and essential role in the management and development of quality. Industry 4.0 allows the company to gain real-time visibility of quality indicators such as production efficiency, supplier performance, engineering manufacturing, and customer support with the aid of ICT developments in Industry 4.0–such as advanced analytics, AI, ML, and IoT [4,72]. A core element of Quality 4.0 is the rapid and efficient data collection from multiple sources to empower informed and agile decision making [70,92,99,101,105].

Analytics: Industry 4.0’s advanced technologies enable us to gather massive data from the production plant and apply the analytics tools to measure the quality matrices. ML and AI insights allow prescriptive analytics to forecast loss and clarify what steps to boost the results [70,92,99,101,105]. 

Connectivity: Quality 4.0 refers to the interaction among information Technology (IT) and operational technology (OT). IT refers to Enterprise Quality Management System (EQMS), Enterprise Resource Planning (ERP), and Product Life Cycle Management (PLM) in this context [72,105]. In contrast, OT refers to technology such as smart devices, sensors, edge devices used in manufacturing plants. Leveraging contact can make it possible to obtain feedback in real-time or near real-time [70,92,99,101,105]. 

Collaboration: Enterprise Quality Management System (EQMS) technologies can allow businesses to optimize and synthesize quality systems to improve compliance and efficiency. Quality 4.0 is designed to leverage modern technology and techniques, such as social listening and blocking, to analyze factors such as customer satisfaction and a more profound sense of component and product distribution across supply chains [105,106].

App development: Apps are valuable tools that help link users and organizations to collect essential data and feedback to enhance services’ quality. Industry 4.0 provides immense promise for designing and developing new applications using augmented reality and virtual reality.

Scalability: Quality 4.0 cannot reconcile procedures, expertise, and best practices fully and efficiently. Industry 4.0’s technologies such as cloud computing, such as software as a service (SaaS), infrastructure as a service (IaaS), or application as a service or platform-as-a-service (PaaS), enables gains in scalability [70,92,99,101,105]. 

Management systems: To benefit from Quality 4.0, organizations must investigate how software automates the process and how those automated processes can be connected to other systems and operations. Improving system autonomy reduces the time that high-value workers and managers spend on implementation and encourages them to focus on improved and innovative jobs [70,92,99,101,105]. 

Compliance: The data collection tasks related to observance can be automated by integrating business information technology and operational technology. The data collection tasks regarding submission can be automated by integrating business information technology and operational technology. Quality 4.0 helps businesses to analyze existing compliance plans and recognize improvement opportunities [70,92,99,101,105]. 

Culture: Quality 4.0, by connecting data, analytics, and processes and improving visibility, connectivity, teamwork, and perspective, allows a real, corporate quality culture more feasible [70,92,99,101,105]. 

Leadership: Quality 4.0 creates the right quality culture throughout the organization more attainable by linking process, information, analytics, and thereby enhancing visibility, communication, Collaboration, and insights [70,92,99,101,105]. 

Competency: Quality 4.0 encapsulates several innovations that can be used to enhance competency. Social media platforms can be leveraged to share lessons and perspectives across organizations and even among organizations. AI and ML systems can create new skills, results from while systems of artificial reality (AR) and virtual reality (VR) can enhance the staff’s expertise [70,92,99,101,105]. In employee assessment, smart devices and wearables can aid when studying management systems, VR and AR can be implemented to enhance training delivery.

Case Study:

In this case study, one of the world’s top aircraft engine manufacturing organizations, Rolls-Royce, is a producer of aircraft engines, supplying more than 150 military aircraft engines and 500 airlines [69]. The manufacturing production plant of Rolls-Royce has been connected, and IoT technology has been applied; the organization uses advanced technologies such as big data to manage aircraft engines and generate a considerable amount of data [70]. Because of the enormous volume of data collected by aircraft engines, ICT technologies for data analysis are built to look at operational strategies to reduce losses by error prevention or failure during the design process [70]. In Rolls-Royce, big data technology is primarily used in three ways: design, manufacturing, and management of sales, in an operating plan that can detect and control the product’s state before problems arise. The nanobots are used for predictive maintenance and inspections at the Rolls-Royce production plant to communicate engine systems better and improve the use of robots where they are dangerous or inaccessible to humans [69,71].

The introduction of this new technological advancement presents an opportunity to improve engine repair strategies by improving the testing process’s speed as part of the maintenance activities or eliminating the need to remove the aircraft’s engine. The Rolls-Royce Company collects data from various sources, such as design, manufacturing, and post-sales management. It analyzes the data collected to generate useful data for predictive maintenance [16] and quality operations. Hundreds of sensors are installed at the Rolls-Royce production plant to collect information and record each small part of the system for a trained staff or supervisor in real-time, which helps the staff or supervisor identify the appropriate actions taken through data analysis. Rolls-Royce presently receives 65,000 h of gas turbine-engine operating data per day with around 100 sensors for pressure, vibration, temperature, velocity, and flow sensors connected to 14,000 engines operated by 500 airlines [69,70,107]. Rolls-Royce offers a post-sale Total Care Service that provides real-time monitoring through data collection [70]. In collaboration with Tata Consultancy services company in India and Microsoft Azure, Rolls-Royce developed a digital platform to connect external information, such as air traffic control, fuel consumption, and weather. The data collected from engine sensors are for glance viewing [2,70]. Before any system failures, these platforms provide predictive maintenance information to airline maintenance teams and passengers with new value-added information, and they allow for a new quality management approach by predictive maintenance [90]. Rolls-Royce can use comprehensive data analysis, intelligent sensors, AI, and platform construction to retain quality control by predictive maintenance. Rolls-Royce shortly predicts the emergence of a business environment where computers, under some conditions, make their own decisions through ML (deep learning).

## 4. Research Challenges, Opportunities, Scope of Future Work and Implication for Practitioners

In this section, we discuss the challenges, opportunities, and scope of the future work of Industry 4.0. With the aid of a questionnaire, we identify the current challenges faced by companies in production systems. Companies are keen to implement innovative innovations to boost resource quality, productivity, and efficiency, reduce risk, and stay competitive [16,108,109,110,111]. A business that struggles to deal with technology complexities also faces implementing new products/services, creativity, and business models, bringing the organization into a fierce competition where expenses have to be reduced each year [112,113,114,115].

It is generally agreed that innovations relevant to Industry 4.0 would significantly affect current industries and future sector development. Although several companies look forward to introducing new technologies to improve their services’ quality, productivity, and efficiency, they reduce risks and sustain market competitiveness [53,116,117,118].

Many challenges need to be addressed in Industry 4.0. In this section, we will discuss a few critical challenges that need to be addressed. In the manufacturing sector, the latest wave of internet technology such as cloud, IoT, big data, robotics, and cyber–physical systems has allowed the manufacturing industry to generate a vast array of business data that will bring new challenges, particularly cybersecurity [79,119,120,121]. These challenges are discussed in Table 5 [49,122,123].

**Table 5:** Challenges and opportunities to over the challenges.

Challenges	Description	References	Opportunities to Overcome the Challenges
Standardization Challenge	Standardization is one of the most critical issues for Industry 4.0 deployment. Difficulties in building up uniform guidelines for data exchange. A reference architecture is necessary for ensuring an interoperable system. It provides a technical overview of the specifications and encourages meaningful cooperation with all users and processes.	[14,94,112,124,125]	Need to Implement uniform standards for information exchange within the organization will help to avoid data loss. Develop a standard protocol for communicating across platforms and ensure it is compatible with our diverse set of tools.
Collaboration Challenge	Collaboration is one of the focus areas in the Industry 4.0 era. Collaboration can occur at different levels within a smart factory and among multiple stakeholders, such as other industries, academic institutions, or business partner In this collaborative environment, solutions will be critical, as they allow access to data not only across plants but across the entire value chain.	[9,41,112,113,126,127,128,129,130,131]	Need to design a collaborative framework. The collaborative framework needs to include coordination, communication and cooperation within the entire organization and stakeholders in the supply chain. The collaboration will bring a new level of end-user experience through socio-technical interaction. The collaboration will help the organization to customize the products as per the end-user requirement. The collaboration will increase the productivity rate in a shorter time.
Cyber Security Challenge	The major concern area of Industry 4.0 is cyber attacks. In the smart manufacturing plant, the shop floor is connected to the internet. Industrial Internet and SCADA systems are appealing targets for cyber-attacks. They control critical infrastructure and processes in manufacturing facilities, power plants, and other industries. An attack can cause damage or even an outage that is expensive to fix.	[123,125,132,133,134,135,136,137]	An organization should launch standardization activities addressing the security of Industry 4.0. An organization should perform an analysis of current security standards to examine whether existing standards adequately address Industry 4.0 security requirements. Need to implement Operation Technology (OT) Security standards -this needs to focus on OT security within the shop floor production. We need to provide training on Cyber security and create awareness about cyber security within the organization.
System Integration Challenge	The integration includes integration of different components, methods and tools, and integration of software and hardware. The first challenge is designing a flexible interface to support different heterogeneous components and supporting adaptive combinations between components. The integration of new technology equipment with existing ones is the key challenge to manufacturing firms. The machine to machine and the interconnection of IT and OT requires a better communication system.	[23,94,95,111,112,125,128,131,138,139,140,141]	It is essential to implement some framework that ensures the security and privacy of production data in order to prevent an attacker from accessing private information. More research work needs to be carried out mainly on the IT and OT integration security-related issue. The integration of the OT and IT will bring many opportunities such as real-time monitoring, customization, smart product, real-time feedback etc.
Communication Challenge	The lack of network connectivity issues	[112,142,143,144,145]	Underdeveloped countries need to establish a good bandwidth network connection throughout the organization.
Environmental Challenges	Industry 4.0 implementation could have serious environmental side effects. For example, companies that rely on automation in the manufacturing process may release high levels of greenhouse gases into the atmosphere. To prevent these effects, companies are challenged with compliance when implementing Industry 4.0.	[9,34,74,146,147,148,149]	Industry 4.0 will change the way people work and live, but there is a risk that this technology could harm the environment. To prevent this from happening, businesses should adhere to environmental standards as they implement Industry 4.0 technologies.

### 4.1. Scope of the Future Work

The definition of the KPI can be further expanded by carrying out studies. The XML implementation of the ISO 22400 Standard, Automation Systems Integration-Key Performance Indicators (KPIs) for Manufacturing Operations Management, is required to implement KPI-ML from MESA. KPI-ML consists of a collection of XML schemas written using the XML Schema Language (XSD) of the World Wide Web Consortium that implements the ISO 22400 standard data models.

### 4.2. Implication for Practitioner

Manufacturing is already on its way to becoming “Industry 4.0”. The findings suggest that the Industry 4.0 initiative will be in high demand in the future. Because Industry 4.0 is a new concept, there is great uncertainty, a lack of knowledge, and little information about performance measurements and quality management in Industry 4.0. Manufacturing companies; conversely, there is still a grappling with the plethora of Industry 4.0 technologies. To close this gap, our research looked at how different industrial standards are used in the manufacturing industry to measure performance and manage product and service quality.

Practitioners can use the study to learn about the various industrial revolutions and how industries are utilizing Industry 4.0 to improve product/process quality and performance.

We discussed the various industrial standards that industries are adopting to bridge the gap between disconnected shop floor production and connected real-time production in this paper. We have shown industrial standards and case studies to show how manufacturing companies are implementing the Industry 4.0 concept to improve overall production plant performance and quality.

Practitioners should use various industrial standards to integrate MES and ERP systems, which will aid in the integration of shop floor production with enterprise systems. They should also look into MESA International’s KPI-ML, an XML version of the ISO 22400 Specifications that will be used to record, interact with, and exchange KPI knowledge.

## 5. Conclusions

This article presents several theoretical and practical models to understand how the data-driven Industry 4.0 or smart manufacturing industries apply the different standards to measure performance and use various frameworks to manage quality. First, the paper described the multiple industrial revolutions based on a comprehensive literature review to understand the digital transformation from the 1960 to 2021. Second, the review discussed the different industrial standards applied for measuring the top-floor level performance in data-driven Industry 4.0. Various standards and case studies used to evaluate the performance of data-driven Industry 4.0 were highlighted and discussed. The ANSI ISA 95 standard focuses on the Manufacturing Operation Center (MOC). The MOC system integrates and creates common ground between the periodic and transactional ERP world suitable for manufacturing plants. Furthermore, it discusses the Overall Equipment Efficiency (OEE) and Analysis of Production loss based on MOC.

The second standard is ISO 22400, which helps to create the new KPI in manufacturing and apply the standards to define the different KPIs to measure the other parameters of performance in smart manufacturing. The review also discussed the most common KPIs used in the industry. It discussed the Scania Pedal Car Line case study to understand how the ISO 22400 standards are being implemented to measure performance. The third section of this review presented quality management and digitalization of quality called Quality 4.0. We discussed the 11 Axes of Quality 4.0, designed by LNS research to understand how Quality 4.0 contributes to better quality. Furthermore, the paper discussed the case study of Rolls-Royce, one of the world’s top three aircraft engine manufacturing companies and how the organization implemented the Industry 4.0 concept to achieve better quality in the competitive market.

Finally, the ISA 95, B2MML, ISO 22400, and KPIML designed by MES need to be examined more systematically, while more organizations implementing the Quality 4.0 framework and how the industries are improving the quality of the product by adopting the Quality 4.0 concept and statistics need to be developed.

## Figures and Tables

**Table 2:** ISO 22400 KPI description table.

KPI Description	
Content	
Name	KPI Name
ID	User-defined unique KPI identification in the user environment
Description	KPI Description in brief
Score	The unit of operation, work center, production order, product, or workers may be the aspect for which the KPI is vital
Formula	For the elements, mathematical formula
Unit measure	The unit or dimension of the KPI
Range	The higher and lower logical limits
Trend	The path of change, higher is better or lower is better
**Context**	
Timing	If the estimate is made in real-time, on-demand, or periodically
Audience	Operators, managers or administrators may be the user Community
Production Methodology	Which methodology can be used for the KPI, discrete, batch or continuous production
Effect Model Diagram	The effect model diagram shows a graphical representation of relationships and dependencies
Notes	

**Table 3:** Most Common KPIs used in industry.

KPI Category	KPI Name	Description
**Improving Quality**	First Pass Yield	This phase indicates the percentage of correctly manufactured products and the specifications for the first time in the manufacturing procedure. Phase without scrapping or rework
**Improving Efficiency**	Throughput Rate	Tests the volume of product Manufactured on a machine, line, unit, or plant over a given period.
**Improving Efficiency**	Availability	Indicates how much of the overall production output is used at a given time. (Included in OEE).
**Improving Efficiency**	Overall equipment efficiency (OEE)	This metric is the Availability × Performance × Quality multiplier and can specify the overall efficacy of production equipment or a production line as a whole.
**Reducing Costs & Increasing Profitability**	Energy consumption	A calculation of the energy costs (electricity, steam, oil, coal, etc.) is needed to produce a particular unit or production volume.

**Table 4:** Visual Process KPIs.

KPI Name	Description
**Count (good or bad)**	This metric refers to the quantity of the finished product. Usually, the count refers to either the amount of product produced after the last changeover of the machine or the total output for the entire shift or week.
**Scrap ratio**	Occasionally, manufacturing processes create scrap, which is calculated in terms of the scrap ratio. Scrap reduction helps organizations achieve profitability goals; thus, controlling the amount generated within tolerable bounds is necessary.
**Throughput Rate**	Machines and processes manufacture products at varying rates. Slow rates usually result in decreased profits as speeds vary, whereas higher speeds influence quality control. This is why staying consistent is critical for operating speeds.
**Target**	Many organizations display performance, rate, and quality target values. This KPI helps empower workers to achieve their specific performance goals.
**Takt Time**	Takt time is the duration of time or the loop. It is also the time to complete a mission.
**Overall Equipment Effectiveness (OEE)**	This metric is the Availability × Performance × Quality multiplier and can indicate the overall efficacy of production equipment or a production line as a whole.
**Downtime**	Downtime is the result of a malfunction or a change of machine. The business can be risky to fail if devices are not running.

## Data Availability

Data sharing is not applicable to this article.
